# Examining an Integrative Cognitive Model of Predicting Health App Use: Longitudinal Observational Study

**DOI:** 10.2196/24539

**Published:** 2021-02-03

**Authors:** Kwanho Kim, Chul-Joo Lee

**Affiliations:** 1 Annenberg School for Communication University of Pennsylvania Philadelphia, PA United States; 2 Department of Communication College of Social Science Seoul National University Seoul Republic of Korea

**Keywords:** mHealth, health apps, digital divide, integrative model of behavioral prediction, path analysis

## Abstract

**Background:**

Specifying the determinants of using health apps has been an important research topic for health scholars as health apps have proliferated during the past decade. Socioeconomic status (SES) has been revealed as a significant determinant of using health apps, but the cognitive mechanisms underlying the relationship between SES and health app use are unknown.

**Objective:**

This study aims to examine the cognitive mechanisms underlying the relationships between SES and use of health apps, applying the integrative model of behavioral prediction (IM). The model hypothesizes the indirect influences of SES on intentions to use health apps, which in turn predict actual use of health apps. The relationships between SES and intentions to use health apps were assumed to be mediated by proximal variables (attitudes, perceived behavioral control [PBC], injunctive norms, and descriptive norms).

**Methods:**

We conducted path analyses using data from a two-wave opt-in panel survey of Korean adults who knew about health apps. The number of respondents was 605 at baseline and 440 at follow-up. We compared our model with two alternative theoretical models based on modified IM to further clarify the roles of determinants of health app use.

**Results:**

Attitudes (β=.220, *P*<.001), PBC (β=.461, *P*<.001), and injunctive norms (β=.186, *P*<.001) were positively associated with intentions to use health apps, which, in turn, were positively related to actual use of health apps (β=.106, *P*=.03). Income was positively associated with intentions to use health apps, and this relationship was mediated by attitudes (B=0.012, 95% CI 0.001-0.023) and PBC (B=0.026, 95% CI 0.004-0.048). Education was positively associated with descriptive norms (β=.078, *P*=.03), but descriptive norms were not significantly related to intentions to use health apps. We also found that PBC interacted with attitudes (B=0.043, SE 0.022, *P*=.046) and jointly influenced intentions to use health apps, whereas the results did not support direct influences of education, income, and PBC on health app use.

**Conclusions:**

We found that PBC over using health apps may be the most important factor in predicting health app use. This suggests the necessity of designing and promoting health apps in a user-friendly way. Our findings also imply that socioeconomic inequalities in using health apps may be reduced by increasing positive attitudes toward, and boosting PBC over, health app use among individuals with low income.

## Introduction

### Overview

Health-related apps (health apps) are software on mobile devices providing various health care services [1,2]. Health apps have been considered new communication technologies that may substantially affect public health [1]. As of 2019, it has been estimated that 54.2% of US adults have health apps [3]; use of health apps can promote prohealth behaviors such as healthy eating [4-6] and weight loss [7,8], though effectiveness of each app may vary [9]. To assess the public health impacts of health apps, scholars have explored predictors of health app use.

Several pioneering studies have reported that demographic factors, including education and income, which are widely used indicators of socioeconomic status (SES), are positively associated with use of health apps [10-13]. Furthermore, some studies examined the roles of SES and cognitive factors as potential determinants of health app use [11,14]. Nevertheless, they did not theorize how SES and cognitive factors are related to each other in predicting health app use. As a result, it is still largely unknown why people with higher SES are more apt to use health apps than those with lower SES.

To address this issue, we propose a comprehensive model of predicting health app use that utilizes the integrative model of behavioral prediction (IM). This theory has been frequently adopted by health researchers to explain the cognitive mechanisms underlying people’s health-related behaviors (eg, safe sex, cancer screening, quitting smoking) [15,16]. We test our model with data from a two-wave panel survey of South Korean adults. Last but not least, to further investigate the relationships between determinants of behaviors in IM, we compare our model with other competing models based on modified IM.

**Applying IM to the Context of Health App Use**

IM succeeds the theory of reasoned action [17] and the theory of planned behavior [18]; all three theories posit that behavioral intention is the primary determinant of a behavior [15,16]. Then, IM theorizes the roles of two different types of variables in predicting behaviors: proximal and distal variables. Only proximal variables directly affect intentions; the influences of distal variables on intentions are mediated by proximal variables.

IM claims that intentions can be sufficiently explained with three proximal variables: (1) attitudes (overall favorableness) toward a behavior, (2) subjective norms regarding a behavior, consisting of injunctive norms (perceptions of what is approved or disapproved by close others) and descriptive norms (perceptions about prevalence of a behavior among close others) on a behavior, and (3) perceived behavioral control (PBC) over a behavior (self-evaluated capability in performing a behavior) [16]. Adopting this argument, we posit hypotheses as follows:

H1-H3: Attitudes toward (H1), subjective norms regarding (H2), and PBC over (H3) health app use will be positively associated with intentions to use health apps, which, in turn, will be associated with increased health app use.

However, resources available for those who attempt to change people’s health-related behaviors are limited. Specifying the proximal variable that most strongly influences a target behavior is necessary to find the most efficient way of affecting it [16]. When it comes to health apps, app developers can devise better promotion strategies and improve the design of their apps more efficiently than they do without such knowledge. For instance, if app developers know that PBC is the strongest determinant of adopting and using health apps, and if the developers have a tight budget, they will want to focus on making apps easy to use. This would be the most cost-efficient way of developing the apps. Hence, we pose the following research question:

RQ1: Which proximal variable will most strongly predict intentions to use health apps?

### Applying IM to Investigate Digital Divide in Using Health Apps

Next, IM categorizes all potential determinants of behaviors other than the proximal variables as distal variables. The relationships between distal variables and intentions are hypothesized to be fully mediated by the proximal variables [16]. Thus, SES is conceptualized as a distal variable in IM. Why, then, does this study concentrate on the relationship between SES and health app use?

Investigating whether and how SES relates to health app use is important in order to know how to reduce the *digital divide* in using the apps. The digital divide refers to inequalities in accessing and utilizing information and communication technologies (ICT) across different social groups [19-22]; this has been revealed as a substantial cause of health disparities [23-25]. Evidence has supported the digital divide in using health apps due to SES [10-13].

Given that cognitive approaches have contributed to understanding the diffusion of ICT [26,27], theorizing the cognitive mechanisms behind the digital divide in using health apps is important to find effective ways of diminishing it.

Nevertheless, former studies have not asked how SES is associated with cognitive factors in predicting health app use. For example, Chae (2018) juxtaposed education and income with cognitive factors in predicting health app use but did not theorize the relationship between SES and cognitive factors [11]; Mackert et al (2016) controlled for demographics when testing the potential connection between health information literacy (ie, cognitive capacity for processing health information) and use of health ICT including health apps [14]. In sum, we propose the following hypothesis:

H4: Individuals’ SES (education and income) will be positively associated with intentions to use health apps through the mediation of proximal variables.

Moreover, in the following research question, we specify the proximal variable that most strongly mediates the influences of SES on behavioral intentions. This will show what will be the most efficient way of decreasing the gaps in use of health apps across people with different SES:

RQ2: Which proximal variable will most strongly mediate the effects of SES on intentions to use health apps?

### Revisiting the Roles of Distal Variables and PBC in IM

Though IM is considered well-established, there are three ongoing controversies regarding the roles of distal variables and PBC in the model [16]. This study will test those competing arguments in the context of health app use.

First, a handful of health studies have found evidence supporting significant *direct* influences of distal variables on behavioral intentions and actual behaviors [28-30]. Those findings confront two fundamental assumptions of IM: (1) indirect relationships between distal variables and intentions and (2) behavioral intention as the primary determinant of behavior. Given those prior findings, we revisit the role of distal variables in the context of health app use as follows:

RQ3: Will SES be directly associated with intentions to use health apps or actual use of health apps?

Next, the original IM argues that the influence of PBC on behaviors is fully mediated by intentions, and it has been consistently supported by evidence [16,31]. However, a few researchers have suggested that PBC may be directly related to behaviors, bypassing the mediation of intentions, to the extent that PBC may reflect actual control over behaviors [31]. Some health studies have reported evidence supporting this competing argument [32-35]. To examine these potential direct influences of PBC on behaviors in the context of health apps, we propose the following research question:

RQ4: Will PBC be directly associated with actual use of health apps?

Lastly, it has been proposed that PBC may moderate the attitudes-intentions and subjective norms–intentions relationships [31,36-38]. The logic of this hypothesis is that positive attitudes and subjective norms may not translate into a behavior when people do not feel that they have sufficient control over (not) conducting the behavior [31,38]. This issue has not been addressed in the context of health apps, and findings from health studies have been mixed. For instance, PBC significantly moderated only attitudes-intentions relationships in the context of prostate-specific antigen testing, whereas only norms-intentions relationships were significantly moderated by PBC in the context of performing regular exercise [37]. Given these mixed findings and the lack of studies addressing this issue in the health app context, we ask the following question:

RQ5: Will PBC moderate attitudes-intentions and subjective norms–intentions relationships in the context of health app use?

## Methods

### Survey Data

This study is a part of a larger health communication research project conducted in South Korea. A two-wave opt-in panel survey of Korean adults was collected by a survey company (Embrain). 1718 respondents participated in the baseline survey in February 2016 (completion rate=1718/2415=71.1%). 1304 of those respondents cooperated with a follow-up survey in April 2016 (attrition rate=414/1718=24.1%). The final sample size decreased to 605 at baseline and 440 at follow-up because we included only those who answered “yes” for the following filter question: “Do you know about health apps? Health app refers to health-related software installed on a smartphone or tablet PC to help users to manage their health behaviors.”

If we had provided a brief explanation about health apps, we could have measured proximal variables and intentions even among the respondents who did not know about the apps. However, measures based on very little knowledge have been considered “tentative”; those should be distinguished from “real” views based on good knowledge about the topic [39]. We adopted the filtered sample that allowed us to focus on those real views about health apps; this increased the validity of our measures and findings [39-41].

When it comes to demographic characteristics, the full (N=1718) and filtered (N=605) samples significantly differed only in years of education (full sample mean 14.97 years, 95% CI 14.86-15.08; filtered sample mean 15.30 years, 95% CI 15.13-15.47). The selected baseline (N=605) and follow-up (N=440) respondents were not significantly different with regard to demographic characteristics. For descriptive statistics, see Table 1.

**Table 1:** Descriptive statistics of variables.

Characteristic	Baseline (n=605)	Follow-up (n=440)
Age (years), mean (SD)	39.00 (10.94)	40.42 (10.81)
Male sex, n (%)	292 (48.3)	210 (47.7)
Employment status (employed), n (%)	431 (71.2)	326 (74.1)
Marital status (married), n (%)	365 (60.3)	284 (64.5)
Years of education, mean (SD)	15.30 (2.14)	15.32 (2.22)
Monthly household income (US $)^a^, mean (SD)	3910.59 (1627.45)	3920.45 (1600.84)
Body mass index	23.10 (3.15)	23.16 (3.02)
Perceived health status^b^, mean (SD)	3.41 (0.75)	3.40 (0.75)
Other source use^c^, mean (SD)	2.64 (0.50)	2.66 (0.50)
Attitudes^d^, mean (SD)	5.01 (0.98)	—^e^
Injunctive norms^f^, mean (SD)	2.69 (0.65)	—
Descriptive norms^g^, mean (SD)	2.01 (0.70)	—
Perceived behavioral control^h^, mean (SD)	3.41 (1.01)	—
Intentions to use health apps^i^, mean (SD)	3.32 (1.07)	—
Types of health apps in use^j^, mean (SD)	2.01 (1.25)	1.80 (1.57)
Frequency of health app use^k^, mean (SD)	2.83 (2.47)	2.50 (2.54)
Health app use (composite measure)^l^, mean (SD)	0.00 (1.56)	0.00 (1.65)

^a^Income was measured on an 8-point scale: 1=US $990 or lower to 8=US $7000 or higher. We averaged household income after recoding the response options into a ratio variable (eg, 2=US $1000 to $1990 was recoded as US $1500).

^b^1=very bad to 5=very good.

^c^Mean of seven items tapping use of health information sources other than health apps. 1=never, 4=frequently.

^d^Mean of two attitudes items. 1=very negative, 7=very positive.

^e^Not available.

^f^1=strongly disagree (low norms), 4=strongly agree (high norms).

^g^1=none of them (low norms), 4=everyone (high norms).

^h^1=no confidence, 5=completely confident.

^i^1=extremely low, 5=extremely high.

^j^Sum of 14 items of a certain type of health app use. 0=no (no use), 1=yes (use).

^k^0=never to 7=everyday.

^l^Sum of the standardized values of frequency of health app use and the number of types of health apps in use.

### Measures

We created survey items capturing IM-related variables in the context of health apps, following the guidelines from Fishbein and Ajzen (2010) [16]. Moreover, we developed a composite measure of health app use following prior studies’ measure creation procedures [42,43].

#### Distal Variables (Baseline)

We adopted education and income as proxies of SES. For education, we asked respondents their highest level of schooling completed (1=elementary school not completed to 8=doctorate). The response options were recoded into years usually required to finish a given type of education in the nation. Income was captured employing an 8-point scale (1=US $990 or lower to 8=US $7000 or higher) and then recoded using the midpoint of each option (eg, 2=US $1000 to $1990 was recoded as US $1500) [44-47].

#### Proximal Variables (Baseline)

To measure attitudes toward using health apps, we averaged participants’ answers to the following two 7-point semantic differential scale items (*r*=0.81): “Using mobile health apps in the next two months would be…” (1=very bad to 7=very good; 1=very unenjoyable to 7=very enjoyable).

We measured injunctive norms by asking respondents about their agreement with the following sentence: “Most people important to me think I should use health apps in the next two months (1=strongly disagree to 4=strongly agree).” Descriptive norms regarding health app use were captured by asking for respondents’ perceptions of how many people important to them had employed health apps in the past two months (1=none of them to 4=everyone). As the correlation between the two norms was only 0.28, they were treated as separate variables in the analyses.

PBC was measured by asking how sure respondents were that they could use health apps on most days in the next two months if they wanted to (1=very unsure to 5=very sure).

#### Intentions to Use Health Apps (Baseline)

To capture intentions to use health apps, we asked participants to report their likelihood of using health apps in the next two months (1=very unlikely to 5=very likely).

#### Health App Use (Baseline & Follow-up)

A composite scale of health app use was constructed by summing the standardized values of the two variables: the frequency of health app use and the number of types of health apps in use (*r*=0.21 at baseline and 0.37 at follow-up). Each reflects the depth and breadth of health app use. The frequency was captured by asking participants about how often they used any health app (0=not at all to 7=all 7 days a week). The other was measured with an additive index of 14 dichotomous items (0=no, 1=yes; the number of users at baseline in parentheses): (a) exercise & fitness (539); (b) healthy diet (145); (c) weight control (141); (d) blood pressure (66); (e) blood sugar (31); (f) menstruation (132); (g) pregnancy (17); (h) baby care (30); (i) medication (12); (j) health information & news (93); (k) mental health (35); (l) sleep (69); (m) quit smoking (20); and (n) beauty (28).

#### Control Variables (Baseline)

We measured demographic variables (age, sex, marital status, employment status, education, and monthly household income) and health-related variables (body mass index, perceived health status). Additionally, we captured use of health information sources other than health apps (hereafter, other source use) by averaging how often respondents obtained health information from the following seven sources (1=never to 4=frequently): printed media, TV, social media, health websites, general websites, friends, and health professionals.

### Analysis Strategy

**Figure 1:**
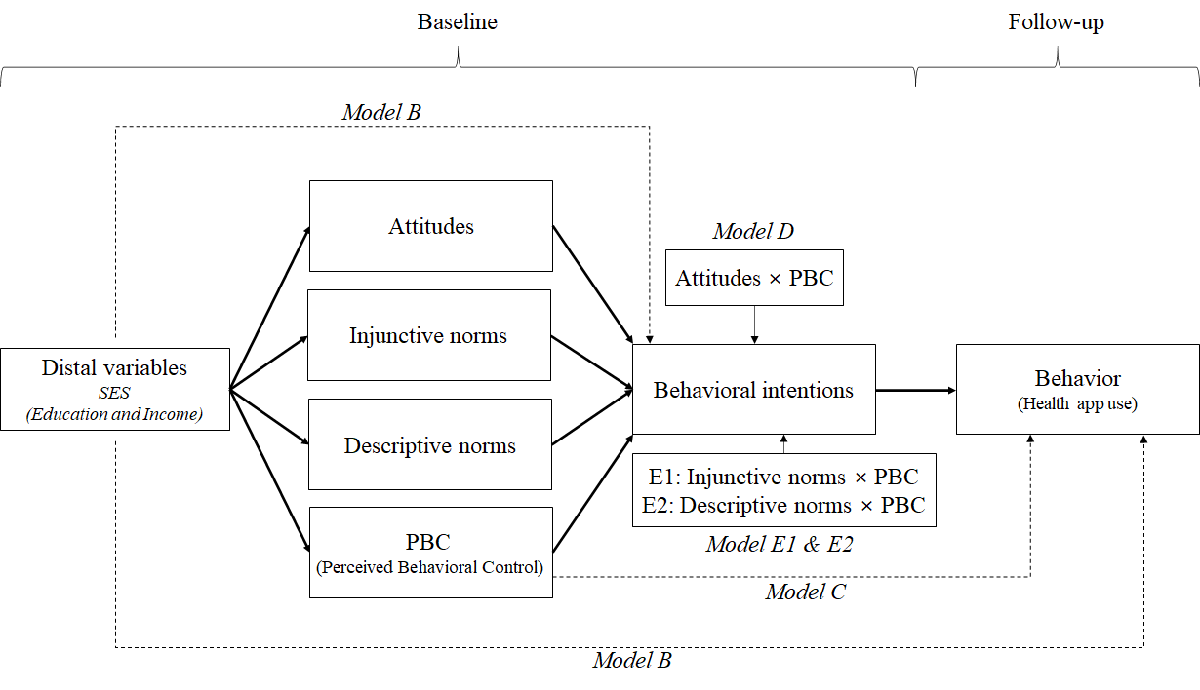
Conceptual framework of the analytic procedure. Bolded solid lines represent paths included in the basic model (Model A). Dashed lines represent paths added to the basic model in each competing model. None of the additional paths were adopted in the final model (Model A). Thin solid lines represent interaction relations added to each of Models D, E1, and E2. SES: socioeconomic status.

We performed path analyses via Mplus 8.3 (Muthén & Muthén). Throughout all analyses, we controlled for all potential confounders described above and health app use at baseline (ie, past behavior). To evaluate model fit, we used a root mean square error of approximation (RMSEA), a comparative fit index (CFI), and a standardized root mean square residual (SRMR): RMSEA≤0.05, CFI≥0.95, and SRMR≤0.08 indicated a well-fitting model [48]. We employed the maximum likelihood with robust standard errors (MLR) method to test direct and interaction effects and bootstrapping (5000 samples) to examine indirect effects. Indirect relationships were considered significant when the bias-corrected 95% CI of unstandardized coefficients did not contain 0 [48]. We adopted the full information maximum likelihood method to handle missing values (aka, FIML and Direct ML). Figure 1 is a graphical illustration of the analysis strategy.

#### The Original IM-based Model

We began by fitting an original IM-based model (hereafter, Model A). In Model A, health app use at follow-up was directly predicted only by intentions at baseline; the associations between intentions and the distal variables (education and income) were fully mediated by proximal variables. We estimated direct and indirect path coefficients to test original IM-based hypotheses (H1 to H4); to compare the relative importance of proximal variables (RQ1 and RQ2), the differences between certain pairs of coefficients were examined with the *χ^2^* difference test. For bivariate correlations of variables in Model A, see Table 2.

**Table 2:** Bivariate correlations of variables in Model A (best-fitting model).

Variable		Education	Income	Attitudes	IN^a^	DN^b^	PBC^c^	Intentions	App use (B)^d^	App use (F)^e^
**Education**										
	*r*	1.000	0.157	0.083	0.052	0.094	0.021	0.086	0.078	0.090
	*P* value	—^f^	<.001	.04	.20	.02	.61	.03	.06	.06
**Income**										
	*r*	0.157	1.000	0.151	0.087	0.078	0.137	0.097	0.122	0.097
	*P* value	<.001	—	<.001	.03	.05	<.001	.02	.003	.04
**Attitudes**										
	*r*	0.083	0.151	1.000	0.482	0.227	0.582	0.584	0.396	0.268
	*P* value	.04	<.001	—	<.001	<.001	<.001	<.001	<.001	<.001
**IN**										
	*r*	0.052	0.087	0.482	1.000	0.275	0.460	0.511	0.321	0.290
	*P* value	.20	.03	<.001	—	<.001	<.001	<.001	<.001	<.001
**DN**										
	*r*	0.094	0.078	0.227	0.275	1.000	0.208	0.224	0.184	0.137
	*P* value	.02	.05	<.001	<.001	—	<.001	<.001	<.001	.004
**PBC**										
	*r*	0.021	0.137	0.582	0.460	0.208	1.000	0.680	0.500	0.336
	*P* value	.61	<.001	<.001	<.001	<.001	—	<.001	<.001	<.001
**Intentions**										
	*r*	0.086	0.097	0.584	0.511	0.224	0.680	1.000	0.581	0.368
	*P* value	.03	.02	<.001	<.001	<.001	<.001	—	<.001	<.001
**App use (B)**										
	*r*	0.078	0.122	0.396	0.321	0.184	0.500	0.581	1.000	0.514
	*P* value	.06	.003	<.001	<.001	<.001	<.001	<.001	—	<.001
**App use (F)**										
	*r*	0.090	0.097	0.268	0.290	0.137	0.336	0.368	0.514	1.000
	*P* value	.06	.04	<.001	<.001	.004	<.001	<.001	<.001	—

^a^IN: injunctive norms.

^b^DN: descriptive norms.

^c^PBC: perceived behavioral control.

^d^B: baseline.

^e^F: follow-up.

^f^Not applicable.

#### Model Comparisons

To address the theoretical controversies about IM, we first created Model B by modifying Model A to include direct links of distal variables (ie, education and income) with intentions and health app use. We compared Models A and B via the *χ^2^* difference test (RQ3). Notably, since we used *χ^2^* values from MLR estimations, the values were first adjusted using the scaling correction factors and then employed for the difference tests. The model fitting the data better was selected and then compared with Model C, constructed by allowing the selected model (Model A or B) to have a direct association between PBC and health app use (RQ4). The better-fitting model in the last comparison was chosen as the final model.

#### Moderating Roles of PBC

To assess the potential moderating roles of PBC (RQ5), we added three mean-centered interaction terms to the final model one at a time (“Attitudes × PBC,” “Injunctive norms × PBC,” and “Descriptive norms × PBC”; Models D, E1, and E2, respectively). We checked the significance of the interaction in each model.

## Results

### The Original IM-based Model

Model A fit the data well (Table 3). We found that intentions at baseline predicted health app use at follow-up (B=0.164, SE 0.075, β=.106, *P*=.03). The effects of attitudes (B=0.241, SE 0.039, β=.220, *P*<.001) (H1), injunctive norms (B=0.307, SE 0.063, β=.186, *P*<.001) (H2), and PBC (B=0.491, SE 0.046, β=.461, *P*<.001) (H3) on intentions were significant, whereas descriptive norms showed no significant association with intentions (B=0.041, SE 0.046, β=.027, *P*=.38) (H2). Accordingly, the indirect effects of attitudes (B=0.040, 95% CI 0.002-0.077) (H1), injunctive norms (B=0.050, 95% CI 0.001-0.102) (H2), and PBC (B=0.081, 95% CI 0.007-0.154) (H3) on follow-up health app use were significant, while the indirect effects of descriptive norms on health app use were not (B=0.007, 95% CI −0.010 to 0.023) (H2). PBC was more strongly related to intentions than were attitudes (*χ*^2^_diff,1_=11.3, *P*<.001) and injunctive norms (*χ*^2^_diff,1_=4.2, *P*=.04). However, the attitudes-intentions relationship was not significantly different from the injunctive norms–intentions relationship (*χ*^2^_diff,1_=0.6, *P*=.43) (RQ1). In sum, H1 and H3 were supported and H2 was partially supported; PBC was revealed as the strongest proximal determinant of intentions to use health apps (RQ1).

**Table 3:** Measures of fit for six models.

Models^a^	Chi-square (*df*)^b^	RMSEA^c^ (90% CI)	CFI^d^	SRMR^e^
Model A	69.2 (35)	0.040 (0.026-0.054)	0.975	0.027
Model B	63.1 (31)	0.041 (0.027-0.056)	0.976	0.025
Model C	67.7 (34)	0.040 (0.026-0.055)	0.975	0.026
Model D	74.7 (37)	0.041 (0.027-0.054)	0.988	0.027
Model E1	70.4 (37)	0.039 (0.024-0.052)	0.975	0.025
Model E2	70.3 (37)	0.039 (0.024-0.052)	0.975	0.025

^a^Model A: the original IM model; Model B: Model A with direct paths of distal variables on intentions and behavior; Model C: Model A with a direct path of perceived behavioral control (PBC) on behavior; Model D: Model A with an interaction term “Attitudes × PBC”; Model E1: Model A with an interaction term “Injunctive norms × PBC”; Model E2: Model A with an interaction term “Descriptive norms × PBC”. Model A was the best-fitting model according to the step-by-step comparisons (step 1: Model A vs Model B; step 2: Model A vs Model C), therefore used for estimating coefficients. Model D, E1 and E2 are employed only for interaction tests.

^b^All *χ*^2^ test results were significant (*P*<.001).

^c^RMSEA: root mean square error of approximation.

^d^CFI: comparative fit index.

^e^SRMR: standardized root mean square residual.

Education was positively associated with descriptive norms (B=0.026, SE 0.012, β=.078, *P*=.03), while income was positively related to attitudes (B=0.050, SE 0.022, β=.096, *P*=.02) and PBC (B=0.053, SE 0.022, β=.100, *P*=.02). The indirect associations between income and intentions through the mediation of attitudes (B=0.012, 95% CI 0.001-0.023) and PBC (B=0.026, 95% CI 0.004-0.048) were significant. However, the indirect relationships between education and intentions through the mediation of descriptive norms were not significant (B=0.001, 95% CI −0.002 to 0.004) (H4). There was no significant difference between the income-attitudes relationship and the income-PBC relationship (*χ*^2^_diff,1_=0.0, *P*=.88) (RQ2). Overall, we found some evidence supporting H4; the importance of proximal variables in predicting intentions to use health apps was not statistically different.

### Model Comparisons

Model A turned out to be the best-fitting model, although Models B and C also fit the data well (Table 3). In the first round of comparison (Model A vs Model B), we found that Model B did not explain the data significantly better than Model A (*χ*^2^_diff,4_=6.1, *P*=.19). That is, direct effects of distal variables on health app use were not supported (RQ3). The winning model, Model A, was further compared with Model C. Still, Model C did not fit the data significantly better than Model A (*χ*^2^_diff,1_=1.4, *P*=.24). In other words, there was no evidence for direct associations of PBC with health app use (RQ4).

### Moderating Roles of PBC

**Figure 2:**
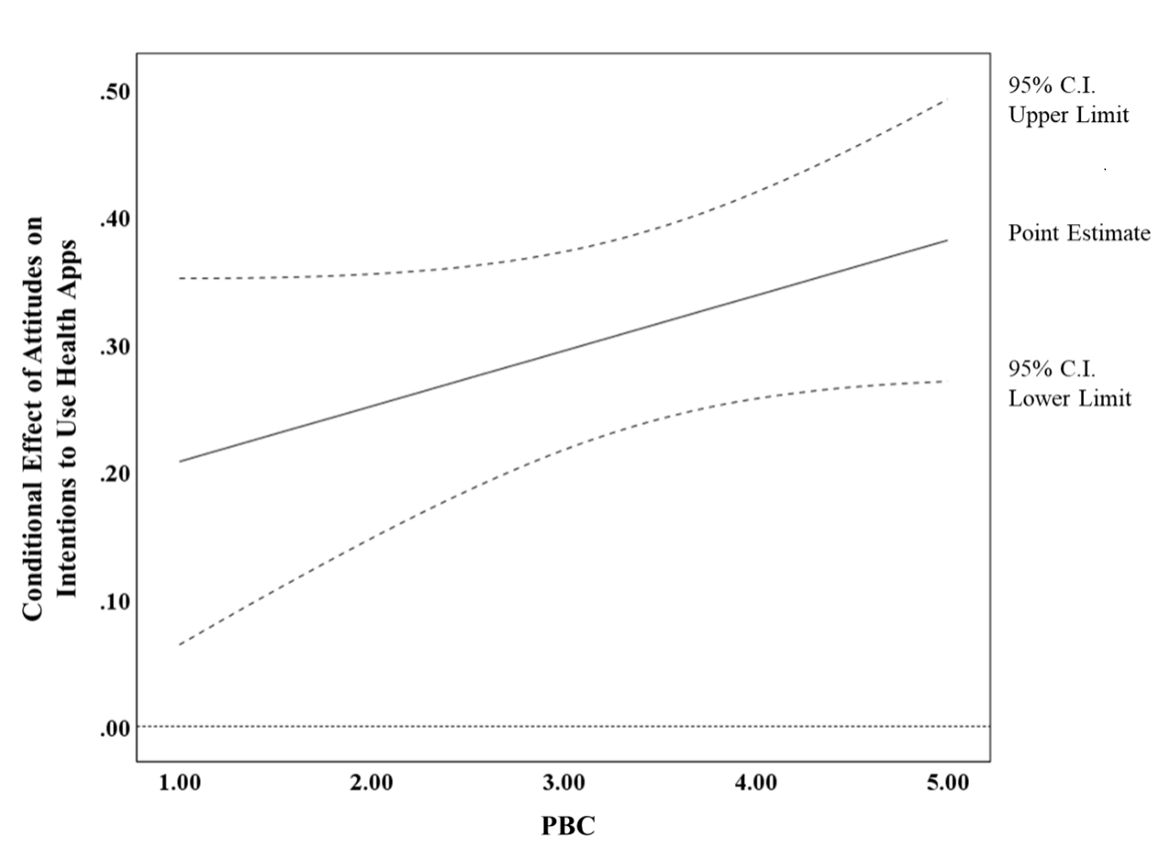
Conditional effect of attitudes on intentions to use health apps as a function of perceived behavioral control from the Johnson-Neyman technique. The 95% CI of the conditional effect of attitudes on intentions to use health apps is always above 0, which means that the effect of attitudes is significantly positive for any value of PBC. PBC: perceived behavioral control.

All models (Models D, E1, and E2) fit the data well (Table 3). The interaction between attitudes and PBC (B=0.043, SE 0.022, *P*=.046) was significant, while neither injunctive norms (B=0.012, SE 0.036, *P*=.75) nor descriptive norms (B=−0.032, SE 0.047, *P*=.49) significantly interacted with PBC in predicting intentions. The results from the Johnson-Neyman technique [49] showed that the 95% CI of the conditional effect of attitudes on intentions was always above 0 (Figure 2). That is, at any range of PBC, the influence of attitudes on intentions was significantly larger for people with higher PBC than for those with lower PBC (RQ5).

## Discussion

As expected, attitudes, PBC, and injunctive norms were associated with intentions, which, in turn, were related to health app use. In contrast, descriptive norms were not significantly related to intentions; thus, they did not affect health app use. PBC positively interacted with attitudes and jointly influenced intention. The association between income and intention was mediated by attitudes and PBC; education was associated with descriptive norms, but the indirect relationship between education and intention was not significant.

Several limitations of this study should be discussed. First, as our data do not represent the Korean adult population, the generalizability of our findings may be restricted. Second, we used single-item questions to measure norms and PBC; future studies should consider employing multiple-item measures. Third, we cannot eliminate the concern of reverse causality because distal and proximal variables and intentions were measured at baseline. Fourth, our health app use measure cannot distinguish people using one app frequently from those who use many apps, but less frequently. However, our measure may better capture the actual pattern of health app use than binary measures (ie, use or no use) adopted in prior studies [11-14,50]. Lastly, future studies may need to control for factors possibly related to both a distal variable and health app use, such as health literacy, which may correlate to SES and health app use.

The theoretical implications of the findings should be highlighted. First, PBC was most strongly associated with intentions. This finding is inconsistent with a well-known argument that subjective norms are the most powerful predictors of behavioral intentions in collectivist cultures, including Korean, while attitudes are key determinants of intentions in individualistic cultures [51]. The relatively low penetration rate of health apps in Korea may explain this discrepancy [52,53].

PBC positively moderated the effects of attitudes on intentions; this has been repeatedly reported in other contexts [36,37,54]. In contrast, the effects of subjective norms on intentions were not moderated by PBC. This finding is not consistent with the prior evidence from Western countries [36,37,54,55]. Perhaps, as subjective norms are more robustly related to behavioral intentions in collectivist cultures than they are in individualistic cultures [51], the relationship might be stable regardless of PBC among Koreans.

We detected significant indirect effects of income on intentions to use health apps. The positive indirect effects of income on intentions mediated by attitudes and PBC were consistent with the key propositions of the diffusion of innovation theory [21]. It argues that individuals with high SES are more likely to be early adopters of technological innovations than people with low SES are, because the former are (1) financially and intellectually more capable of employing new technologies (ie, higher PBC) and (b) more open-minded to use those technologies than are the latter (ie, higher positive attitudes).

The findings of this study also have the following practical implications: First, this study underscores the importance of PBC in designing and promoting health app use. We discovered that PBC was the strongest determinant of intentions to use health apps and moderated the influences of attitudes on intentions. To boost PBC, an app should be designed and promoted in a user-friendly way (eg, using plain and easy-to-read language; providing easy-to-follow guidelines) so that potential users will not experience difficulties in using the app. Second, this study suggests that, to reduce the digital divide in health app use, public health professionals should instill in low-income individuals beliefs about expected positive outcomes from, and confidence in, using health apps. This strategy would thereby form favorable attitudes toward and greater PBC over health app use. Health apps are frequently monetized; thus, they are designed to target people with high SES to maximize their developers’ profits [56,57]; given our findings, this trend is particularly worrisome because it can maintain or even worsen inequalities in public health outcomes.

## Abbreviations

CFI: comparative fit index
ICT: information and communication technology
IM: integrative model of behavioral prediction
MLR: maximum likelihood with robust standard errors
PBC: perceived behavioral control
RMSEA: root mean square error of approximation
SES: socioeconomic status
SRMR: standardized root mean square residual
